# Diffusion levels for quantitative assessment of the apparent diffusion coefficient value in prostate MRI: a proof-of-concept bicentric study

**DOI:** 10.1007/s00330-025-11547-8

**Published:** 2025-04-07

**Authors:** Rossano Girometti, Valeria Peruzzi, Paola Clauser, Nina Pötsch, Maria De Martino, Miriam Isola, Gianluca Giannarini, Alessandro Crestani, Chiara Zuiani, Lorenzo Cereser, Pascal AT Baltzer

**Affiliations:** 1 Institute of Radiology, Department of Medicine (DMED), University of Udine, Udine, Italy; 2 Azienda Sanitaria Universitaria Friuli Centrale (ASUFC), University Hopital “S. Maria della Misericordia”, Udine, Italy; 3 Department of Biomedical Imaging and Image-guided Treatment, Medical University of Vienna, Vienna, Austria; 4 Division of Medical Statistics, Department of Medicine (DMED), University of Udine, Udine, Italy; 5 Urology Unit, Azienda Sanitaria Universitaria Friuli Centrale (ASUFC), University Hopital “S. Maria della Misericordia”, Udine, Italy; 6 Urology Unit, Department of Medicine (DMED), University of Udine, Udine, Italy

**Keywords:** Prostatic neoplasms, Magnetic resonance imaging, Diffusion magnetic resonance imaging, Biopsy

## Abstract

**Objectives:**

To investigate the performance of Diffusion levels (DLs) in diagnosing clinically significant prostate cancer (csPCa) when combined with the PI-RADS version 2.1.

**Materials and methods:**

This retrospective, bicentric study included 261 men who underwent 3.0-T prostate MRI between March 2020 and April 2023, receiving systematic and target prostate biopsy on PI-RADS ≥ 3 lesions. Two readers measured the Apparent diffusion coefficient (ADC) of PI-RADS 1–5 findings in the peripheral zone. By plotting the cumulative frequency of csPCa versus ADCs and using ROC analysis, we derived four DLs expressing levels of restricted diffusion, i.e., very low DL (VL-DL), low DL (L-DL), intermediate DL (I-DL), and high DL (H-DL). We compared the per-lesion diagnostic performance in assessing csPCa (grading group ≥ 2 cancer) assuming to biopsy PI-RADS ≥ 3 lesions (strategy 1), PI-RADS ≥ 3 lesions adjusted with ADC values (strategy 2–4), and PI-RADS ≥ 3 lesions adjusted with DLs (strategy 5–7). Net benefit was assessed with decision curve analysis.

**Results:**

csPCa was found in 79/261 men (30.3%) and 152/528 lesions (28.8%). There was a negative correlation (*p* < 0.0001) between ADC versus malignancy rate (tau −0.970) and DLs versus csPCa grading group (tau −0.614). csPCa prevalence was highest in VL-DL (72.2%) and L-DL (54.4%). Most DLs-based strategies increased specificity, positive predictive value (PPV), and net benefit compared to ADC-based strategies or PI-RADS alone. The best strategy showed 94.7% sensitivity, 82.9% specificity, 69.2% PPV, and 97.5% negative predictive value.

**Conclusion:**

While larger-scale validation is needed, DLs have the potential to improve PI-RADS-based biopsy decisions for detecting csPCa in the peripheral zone.

**Key Points:**

***Question*** *It is still unclear how to incorporate quantitative information from diffusion-weighted imaging (DWI) into prostate MRI.*

***Findings*** *Combining DWI-derived diffusion levels (DLs) with the PI-RADS version 2.1 categorization reduced false positives while preserving high sensitivity for clinically significant prostate cancer.*

***Clinical relevance*** *DLs permit to easily account for ADC values of prostate lesions and, in turn, refine biopsy decisions.*

**Graphical Abstract:**

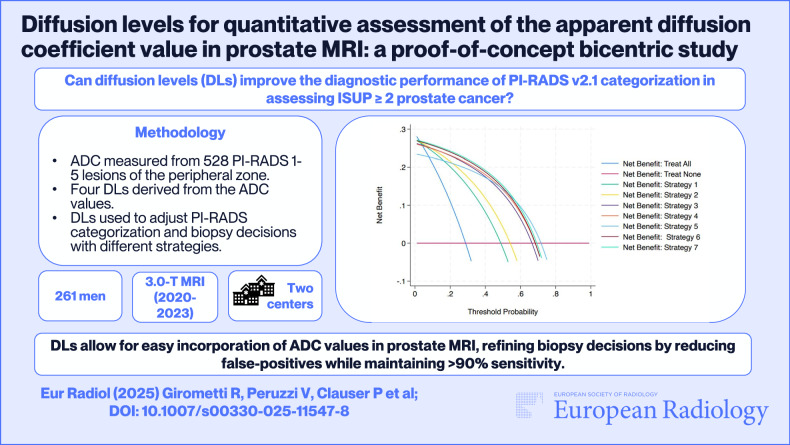

## Introduction

According to the Prostate imaging reporting and data system (PI-RADS) [1, 2], Diffusion-weighted imaging (DWI) is a crucial component of MRI of the prostate, having a dominant role in establishing the PI-RADS category of lesions located in the peripheral zone (PZ). This, in turn, translates into a pivotal impact on biopsy decisions and the selection of targets for MRI-informed prostate biopsy [3].

Despite expectations of the apparent diffusion coefficient (ADC) as a potential biomarker in diagnosis, active surveillance, and risk stratification of prostate cancer [4], the current use of DWI relies only on visual assessment of restricted diffusion on the ADC map [2]. Although PI-RADS version 2 suggested ADC values of 0.75–0.90 × 10^–3^ mm²/s to assist in differentiating benign from malignant prostate tissues in the PZ [1], a standardized approach to quantify DWI reliably remains elusive due to variability in ADC measurements across readers, cohorts, MRI machines, coils, and protocols, as well as challenges in defining an absolute malignancy cutoff [5–7].

The “ADC ratio” between lesion ADC and a reference tissue showed promising results to overcome these limitations [8–10]. However, placing a region of interest (ROI) over a reference tissue such as the bladder wall, urine, pelvic muscles [5], or even the “normal prostate” in men affected by benign prostatic hyperplasia or active prostatitis can lead to difficult-to-reproduce results. Several studies showed no added value from using the ADC ratio [11–14].

A potential solution for implementing ADCs in prostate MRI interpretation comes from a different clinical setting. In 2020, a consensus and mission statement from the DWI working group of the European Society of Breast Imaging (EUSOBI) proposed technical recommendations and interpretation rules to promote DWI in breast MRI [15]. The working group defined five different ranges of ADC values derived from a meta-analysis [16] and assumed they correspond to as many diffusion levels (DLs) (very low, low, intermediate, high, and very high) reflecting different risks of malignancy and lesion types. This strategy overcomes the need for an absolute threshold of malignancy and improves diagnostic performance in assessing breast cancer by complementing the Breast Imaging reporting and data system [17]. Bickel et al [18] confirmed the potential for a reproducible and accurate use of the ADC by building DLs from a large dataset of real-world breast MRIs across different centers.

As far as we know, no previous studies assessed whether DLs can be derived from prostate MRI and whether they can reliably correspond to the risk of clinically significant prostate cancer (csPCa) or different International Society of Urological Pathology (ISUP) grading groups. If so, DLs could represent a strategy to standardize the interpretation of prostate lesions ADC and, in turn, complement the PI-RADS and refine biopsy decisions accordingly.

We aimed to build DLs from a bicentric dataset of real-world prostate MRI examinations and test their diagnostic performance in assessing csPCa as a stand-alone tool or combined with the PI-RADS version 2.1. Analysis was focused on PZ lesions since we assumed that DLs should be preliminary tested in the prostate zone where DWI is dominant.

## Materials and methods

### Study population

The Institutional Review Board of Center 1 approved the study and granted a waiver for acquiring patient informed consent. In this institution, ADC measurements described below were performed de novo for the purpose of the study. In Center 2, a fully anonymized database of ADC values of prostate lesions was already collected for the purpose of different research, with use for any future retrospective study granted by local Ethical Review Board approval and previously acquired written informed consent. The period of patient inclusion was March 2020–September 2022 in Center 1 and May 2021–April 2023 in Center 2.

**Fig. 1:**
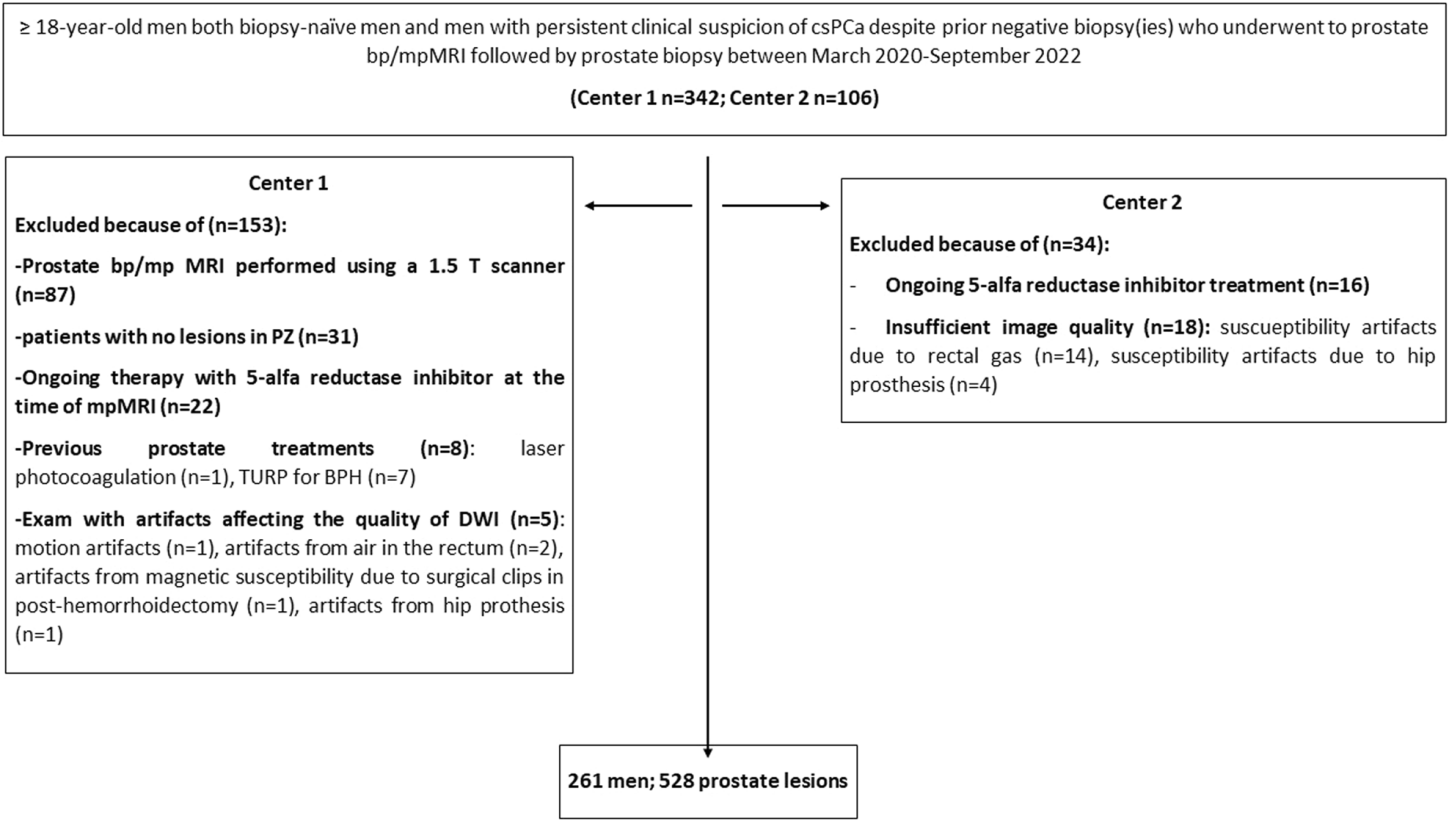
Study flowchart. No men were excluded because of the absence of measurable findings in Center 2. *BPH* benign prostatic hyperplasia, *bpMRI* biparametric magnetic resonance imaging, *mpMRI* multiparametric magnetic resonance imaging, *PZ* peripheral zone, *TURP* Transurethral resection of the prostate

In both institutions, we included ≥ 18-year-old men who consecutively underwent MRI followed by prostate biopsy because of at least one PI-RADS ≥ 3 lesion or higher clinical risk despite a negative examination (PI-RADS ≤ 2). Indications of MRI were increased prostatic specific antigen (PSA) serum level (≥ 3.0 ng/mL in two serial samples) and/or suspicious digital rectal examination (DRE). All the included men were of Caucasian ethnicity. Criteria for auctioning prostate biopsy in men with negative MRI were not standardized but rather at the urologists’ discretion based on PSA and PSA density values, PSA kinetics, DRE, family history, and prior negative biopsy, if any. Exclusion criteria are illustrated in the study flowchart (Fig. 1). Ongoing therapy with 5-alfa reductase inhibitors was among the exclusion criteria to prevent reduced conspicuity on high b-value images [19].

### Standard of reference

All the included men received 12-core systematic prostate biopsy plus target biopsy on all the suspicious MRI findings (PI-RADS ≥ 3) prompted in the original MRI report. Lesions reported as PI-RADS 1 or 2 underwent target biopsy in no cases. Additional details on the biopsy procedure are shown in the Supplementary Material. Histological analysis of the biopsy samples by referring uropathologists was the standard of reference for csPCa, which was assumed to be an ISUP grading group ≥ 2 cancer [20].

Target biopsy included four cores (two in-target and two perilesional cores) in Center 1 and Center 2. In both centers, the procedure was performed under local anesthesia by one of a pool of experienced urologists, using fusion ultrasound-mpMRI guidance (Applio 300 platform, Toshiba/Canon in Center 1; Uronav system by Philips Healthcare in Center 2). The biopsy route was transperineal in Center 1 and transrectal in Center 2.

### MRI examinations

MRI examinations were performed on one of several 3.0-T magnets (Achieva, Philips Medical Systems, in Center 1 and several 3-T units, mostly Prisma fit, Siemens Healthineers, in Center 2) using a surface coil.

Both centers utilized a dual DWI sequence approach, using the vendor’s software to perform a linear regression of signal intensity versus the b-values of the first DWI sequence with a maximum b of at least 1000 s/mm^2^ to generate the ADC map. Technical acquisition details are shown in Supplementary Tables 1 and 2.

### ADC measurements, DLs building, and biopsy strategies

In Center 1, one reader qualified as an expert according to reference criteria [21] replicated the image analysis strategy previously adopted in Center 2 by one different radiologist with comparable experience. Center 1 readings were performed retrospectively compared to MRI and prostate biopsy. Readers were blinded to clinical history and biopsy results but not to the MRI report and were asked to measure the ADC of any previously reported PI-RADS 1–5 lesion in the PZ using a dedicated console (SuitEstensa, Esaote, in Center 1 and Impax, Agfa HealthCare, in Center 2). In both centers, previously reported lesions were identified for ADC measurement using the PI-RADS sectorial map and key images appended to the description made in the original report. Reflecting the EUSOBI principles to obtain the DLs [15], the ADC was measured by placing the largest possible region of interest (ROI) on the most hypointense part of a lesion in the ADC map, having care this corresponded to a visible signal alteration on the high b-value image and/or on dynamic contrast-enhanced imaging, if any. In the case of uncertainty about the most hypointense part of the lesion, multiple ROIs were placed, and the one with the lowest ADC value was selected for analysis. Notably, MRI examinations classified as PI-RADS 1 showing no focal lesions at all in the PZ were excluded from analysis (Fig. 1), based on the assumption that DLs have limited significance in the absence of measurable findings. Thus, ADC was measured on “PI-RADS 1” measurable lesions, reported as such because of their clearly benign appearance (e.g., ectopic nodules of the transition zone). This led to build DLs over the whole spectrum of measurable lesions.

DLs were derived from the ADC values by modifying the step-by-step methodology used by Bickel et al [18]. First, we plotted all the collected ADC values against the cumulative frequency of csPCa, testing the correlation with Kendall’s tau index. Second, we run a receiving operating characteristics (ROC) analysis to identify the ascending ADC value ranges corresponding to the following intervals of sensitivity and specificity in assessing csPCa, which were, in turn, assumed to represent as many DLs: sensitivity < 60% and specificity > 90% (very low diffusion level (VL-DL)); sensitivity 60–75% and specificity 85–90% (low diffusion level (L-DL)); sensitivity 75–95% and specificity 70–85% (intermediate diffusion level (I-DL)); sensitivity > 95% and specificity ≤ 70% (high diffusion level (H-DL)). We then calculated the actual prevalence of benign lesions, ISUP ≥ 1 cancers, and ISUP ≥ 2 cancers on a per-DLs basis and used Kendall’s tau index to assess the correlation between DLs and the observed ISUP grading groups.

The ADC threshold found at ROC analysis according to the Youden index was used in further analysis as the “absolute ADC cutoff” mentioned below.

### Data analysis

As the Shapiro-Wilk test showed a non-normal distribution of continuous variables, they were summarized by reporting the median values with the interquartile range (IQR). However, we also showed mean ± standard deviation values to facilitate the comparison with previous literature. The Kruskal-Wallis test was used to compare the ADC values across benign lesions, ISUP 1 and ISUP ≥ 2 cancers, using the u Mann–Whitney test for pairwise comparisons. The alfa level was 0.05 with the Bonferroni correction when applicable.

**Fig. 2:**
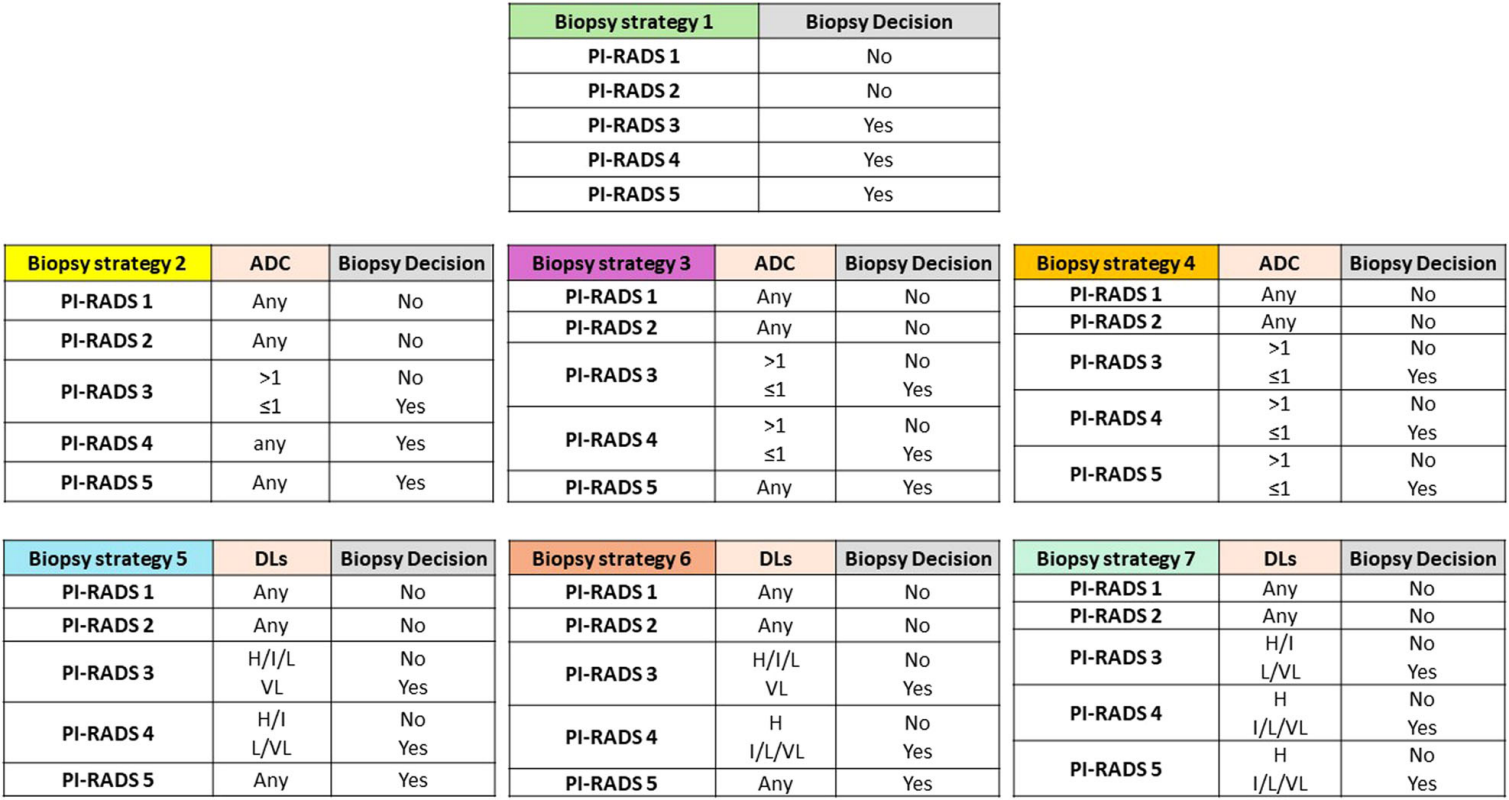
Biopsy decisions based on PI-RADS version 2.1 alone (strategy 1), PI-RADS version 2.1 adjusted with an absolute apparent diffusion coefficient (ADC) cutoff of 1.0 × 10^−^^3^ mm^2^/s (strategy 2 to 4), and PI-RADS version 2.1 adjusted with the Diffusion Levels (DLs)

To assess whether DLs improve the diagnostic performance of MRI-informed biopsy decisions, we compared six different biopsy strategies against strategy 1, i.e., sampling all PI-RADS ≥ 3 lesions (Fig. 2). Biopsy strategies 2–4 were supposed to adjust the PI-RADS categorization with the absolute ADC cutoff found at ROC analysis. In contrast, strategies 5–7 were supposed to adjust the PI-RADS categorization with DLs. We calculated the sensitivity, specificity, positive predictive value (PPV), and negative predictive value (NPV) for csPCa at a lesion-level, assuming a fixed comparable threshold for auctioning prostate biopsy, i.e., PI-RADS 3 category (strategy 1) or PI-RADS 3-adjusted category (strategies 2–7). Biopsy targets above the threshold were categorized as “true positive” if csPCa was found in at least one core of the target biopsy or the systematic biopsy performed in an adjacent quadrant. Biopsy targets below the threshold were defined as “true negative” if there was no csPCa in any target biopsy core or systematic biopsy core from the adjacent quadrants. Given the assumption of a fixed threshold for biopsy, we did not run ROC analysis to compare the strategies.

Finally, we used decision curve analysis to establish which biopsy strategy showed the greatest clinical utility in terms of net benefit, i.e., the advantage of detecting true positives adjusted for the harm of false positives against a “treat none” strategy (biopsying no lesions) and a “treat all” strategy (biopsying all lesions).

## Results

### mpMRI findings and biopsy results

The final population included 261 men with 528 PI-RADS 1–5 lesions overall (Fig. 1). Men had a median (IQR) age of 65.0 years (59.0–71.0), a PSA level of 6.19 ng/mL (4.72–8.89), and a PSA density of 0.12 ng/mL^2^ (0.08–0.18). Of them, 230/261 (88.1%) were biopsy-naïve, while 31/261 (11.9%) had a prior negative biopsy. Except for 2 out of 261 cases in Center 1 where contrast administration was contraindicated, all men underwent multiparametric MRI.

Lesions characteristics are summarized in Table 1. ISUP ≥ 2 cancer was found in 79/261 men (30.3%; 95% CI 23.9–37.7) and 152/528 lesions (28.8%; 95% CI 24.4–33.8). Supplementary Table 3 shows data stratification on a per-center basis.

**Table 1:** Lesions’ characteristics on magnetic resonance imaging (MRI) and prostate biopsy

MRI and histological features	Median value (IQR)	Prevalence (%)	Prevalence of csPCa (%)
PI-RADS version 2.1 category			
1		48/528 (9.1%)	0/48 (0%)
2	-	185/528 (35.0%)	8/185 (4.3%)
3	-	40/528 (7.6%)	4/40 (10%)
4	-	175/528 (33.1%)	86/175 (49.1%)
5	-	80/528 (15.2%)	54/80 (67.5%)
ISUP grading group			
No cancer	-	285/528 (54%)	-
1	-	91/528 (17.2%)	-
2	-	72/528 (13.6%)	-
3	-	44/528 (8.3%)	-
4	-	17/528 (3.2%)	-
5	-	19/528 (3.6%)	-

*csPCa* clinically significant prostate cancer, *IQR* interquartile range, *ISUP* International Society of Urological Pathology, *PSA* prostatic specific antigen

### ADC values and DLs

Table 2 and Supplementary Fig. 1 show mean and median ADC values measured across all the ROIs and lesion histology. The ADC values were significantly higher in benign lesions versus ISUP 1 or ISUP ≥ 2 cancers, as well as in ISUP 1 versus ISUP ≥ 2 cancers (*p* < 0.0001 for any comparison). Even the ADC values of benign lesions plus ISUP 1 cancers were significantly higher than those of ISUP ≥ 2 cancers (*p* = 0.0002).

**Table 2:** The apparent diffusion coefficient (ADC) values according to prostate biopsy

	ADC values (× 10^−^^3^ mm^2^/s)				
ROIs	Mean	Standard deviation	Median	IQR	Range
All	1.10	0.38	1.06	0.83–1.32	0.18–2.28
Benign lesions	1.32	0.34	1.29	1.09–1.52	0.54–2.17
ISUP 1			1.01	0.85–1.14	0.34–2.28
Benign lesions and ISUP 1	1.24	0.35	1.20	1.01–1.44	0.34–2.28
ISUP 2	0.88	0.23	0.87	0.74–0.96	0.45–1.79
ISUP 3	0.70	0.13	0.69	0.63–0.79	0.33–1.00
ISUP 4	0.64	0.19	0.65	0.50–0.80	0.20–0.97
ISUP 5	0.66	0.24	0.67	0.60–0.83	0.18–0.99
ISUP ≥ 2	0.77	0.23	0.77	0.64–0.89	0.18–1.79

*IQR* interquartile range, *ISUP* International Society of Urological Pathology, *ROIs* regions of interest

**Fig. 3:**
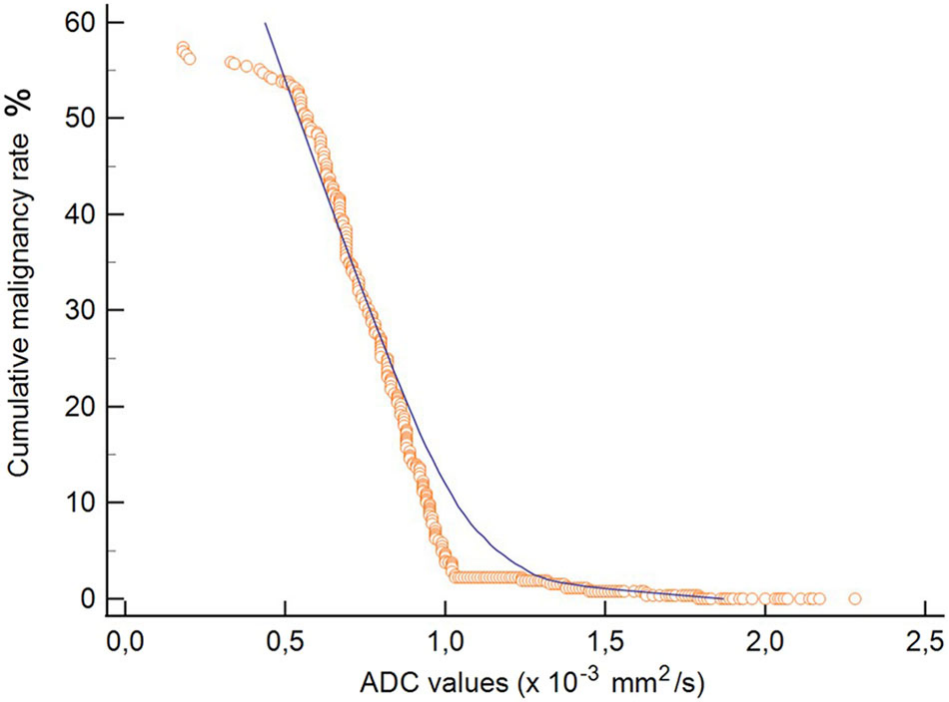
Cumulative malignancy rates (*y*-axis) plotted against apparent diffusion coefficient (ADC) values (*x*-axis). Dots on the curve represent each single prostate lesion. The malignancy rate values corresponding to each diffusion level were ≤ 2.4% for the high level, 2.8–13.6% for the intermediate level, 13.6–22.9% for the low level, and 23.1–57.4% for the very low level

The cumulative malignancy rate decreased with a strong negative correlation with ascending ADC values (tau = −0.970, *p* < 0.0001). The relationship was steeply almost linear for ADC values below 1.03 × 10^−^^3^ mm^2^/s (Fig. 3).

Table 3 shows the DLs we derived from ROC analysis (plot in Supplementary Fig. 2). The absolute ADC cutoff for csPCa was ≤ 1.0 × 10^−^^3^ mm^2^/s, corresponding to a sensitivity of 93.4% (95% CI 88.2–96.8) and specificity of 75.5% (95% CI 70.9–79.8). VL-DL more frequently corresponded to ISUP ≥ 2 (91/126, 72.2%), especially ISUP ≥ 3 cancers (64/126, 50.8%). The L-DL showed greater dispersion across different ISUP grading groups. The overall prevalence of ISUP ≥ 2 cancers was 54.4% (25/46), most of which were represented by ISUP 2 lesions (16/25 lesions, 64%). H-DL and I-DL prevalently included benign lesions (229/280, 81.8%) and benign lesions or ISUP 1 cancers (47/76, 61.8%), respectively.

**Table 3:** DLs used in the study to refine biopsy decisions

Diffusion levels (DLs)	Corresponding ADC range (× 10^−^^3^ mm^2^/s)	Prevalence of benign lesions (%; 95% CI)	Prevalence of ISUP ≥ 1 cancers (%; 95% CI)	Prevalence of ISUP ≥ 2 (%; 95% CI)
Very low diffusion level (VL-DL)	0.18–0.82	15/126 (11.9; 6.7–19.6)	111/126 (88.1; 72.5–100)	91/126 (72.2; 58.2–88.7)
Low diffusion level (L-DL)	0.83–0.91	11/46 (23.9; 11.9–42.8)	35/46 (76.1; 53.0–100)	25/46 (54.4; 35.2–80.2)
Intermediate diffusion level (I-DL)	0.92–1.02	30/76 (39.5; 26.6–56.4)	46/76 (60.5; 44.3–80.7)	29/76 (38.2; 25.6–54.8)
High diffusion level (H-DL)	1.03–2.28	229/280 (81.8; 71.5–93.1)	51/280 (18.2; 13.6–23.9)	7/280 (2.5; 1.0–5.2)

Clinically significant cancer was assumed to be grading group ISUP (International Society of Urological Pathology) 2 or larger

*ADC* apparent diffusion coefficient

H-DL cases were mostly categorized PI-RADS 1–2 (198/280; 70.7%), while most I-DL, L-DL, and VL-DL cases were categorized PI-RADS ≥ 3 (54/76, 71.1%; 40/46, 87.0%; 118/125, 94.4%, respectively) or PI-RADS ≥ 4 (49/76, 64.4%; 36/46, 78.2% and 114/126, 90.4% respectively). Supplementary Tables 4 and 5 detail the prevalence of benign lesions and different ISUP grading groups on a per-DLs basis or the PI-RADS categorization. There was a significantly negative correlation (*p* < 0.0001) between DLs and the ISUP grade (tau = −0.614; 95% CI −0.660 to −0.555).

### Diagnostic performance and net benefit

**Fig. 4:**
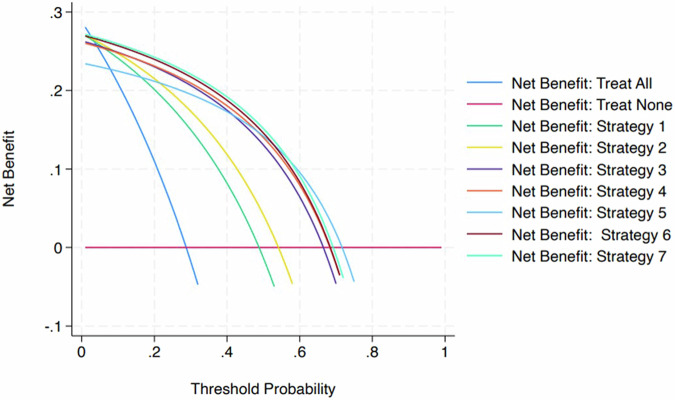
Results of the decision curve analysis plotting the net benefit of performing prostate biopsy against the probability of having clinically significant prostate cancer according to seven different biopsy strategies (see Fig. 2 for details)

**Fig. 5:**
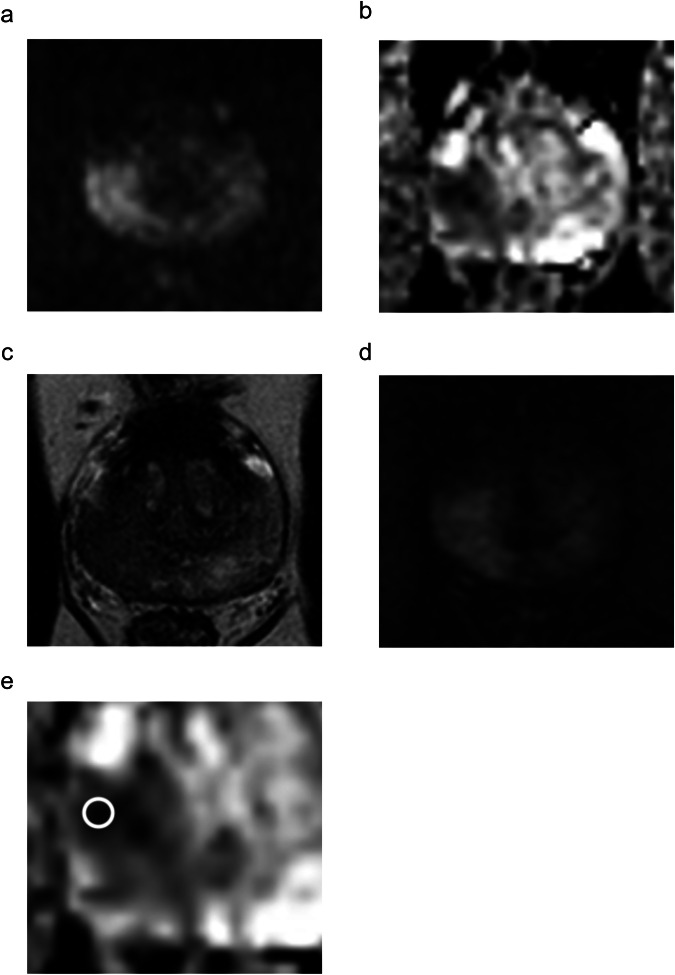
A 56-year-old man showing a prostate-specific antigen level of 21.2 ng/mL and a PI-RADS 5 lesion in the right postero-lateral zone of the mid-gland, corresponding to a grading group 3 cancer. The lesion showed marked hyperintensity on b = 2000 s/mm^2^ b-value image (**a**), marked hypointensity on the ADC map (**b**), hypointensity on T2-weighted imaging (**c**), and intense early focal contrast enhancement (**d**). In accordance with the criteria explained in the “Materials and methods,” the region of interest was placed on the darkest region of the lesion on the ADC map (zoom of the lesion in **e**), measuring an apparent diffusion coefficient value of 0.65 × 10^−^^3^ mm^2^/s, corresponding to a very low diffusion level

**Fig. 6:**
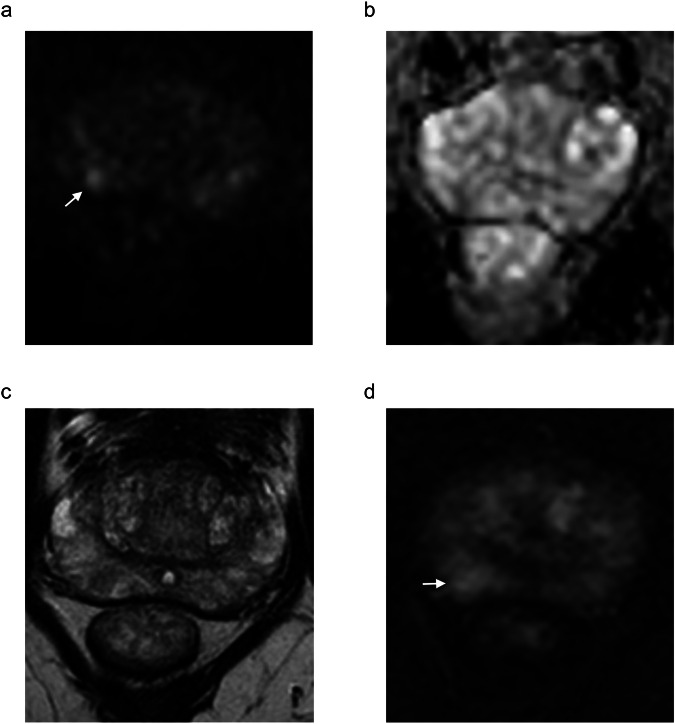
A 60-year-old man showing a PSA level of 4.45 ng/mL. PI-RADS 3 upgraded to 4 category was attributed to a lesion in the right mid-gland posterior-lateral peripheral zone showing marked hyperintensity on the high b-value image (arrow in **a**), mild hypointensity on the ADC map (**b**), mild hypointensity on T2-weighted imaging (**c**) and focal early contrast enhancement (arrow in **d**). Target biopsy cores included high-grade prostatic intraepithelial neoplasia, atypical small acinar proliferation, and chronic inflammation. Biopsy strategy 7 would have avoided biopsy as the lesion showed an ADC value of 1.11 × 10^−^^3^ mm^2^/s, corresponding to a high diffusion level (H-DL)

The diagnostic performance of different biopsy strategies is shown in Table 4, while the decision curve analysis plot and net benefit values are reported in Fig. 4 and Table 5. Example cases are shown in Figs. 5 and 6. Overall, strategy 7 showed the highest net benefit values at reference probabilities, i.e., 0.593, 0.2424, 0.2208, and 0.1919 at 10%, 20%, 30% and 40% probability of csPCa, respectively.

**Table 4:** Diagnostic performance of the biopsy strategies in detecting clinically significant prostate cancer

Biopsy strategy	Sensitivity % (95% CI)	Specificity % (95% CI)	PPV % (95% CI)	NPV % (95% CI)	TP/TN/FN/FP (number)
1	94.7 (89.9–97.7)	59.8 (54.7–64.8)	55.6 (46.9–65.4)	96.6 (84.3–100)	144/225/8/151
2	94.7 (89.9–97.7)	67.5 (62.6–72.3)	54.1 (45.6–63.7)	96.9 (85.4–96.3)	144/254/8/122
3	91.4 (85.8–95.4)	81.4 (77.1–85.2)	66.5 (55.9–78.5)	95.9 (85.5–100)	139/306/13/70
4	90.8 (85.0–94.9)	82.9 (78.8–86.6)	68.3 (57.4–80.7)	95.7 (85.4–100)	138/312/14/64
5	81.6 (74.5–87.4)	86.9 (83.1–90.2)	71.7 (59.6–85.4)	92.1 (82.4–100)	124/327/28/49
6	94.1 (89.1–97.3)	82.4 (78.2–86.2)	68.4 (57.6–80.6)	97.2 (86.7–100)	143/310/9/66
7	94.7 (89.9–97.7)	82.9 (78.8–86.6)	69.2 (58.4–81.5)	97.5 (86.9–100)	144/312/8/64

*AUC* area under the curve at the receiver operating characteristics analysis

**Table 5:** Net benefit of the biopsy strategies in detecting clinically significant prostate cancer, which was assumed to be grading group ISUP (International Society of Urological Pathology) 2 or larger

	Net benefit			
Biopsy strategies	10%	20%	30%	40%
Biopsy strategy 1	0.2410	0.2012	0.1502	0.0821
Biopsy strategy 2	0.2471	0.2150	0.1737	0.1187
Biopsy strategy 3	0.2485	0.2301	0.2064	0.1749
Biopsy strategy 4	0.2479	0.2311	0.2094	0.1806
Biopsy strategy 5	0.2245	0.2116	0.1951	0.1730
Biopsy strategy 6	0.2569	0.2396	0.2173	0.1875
Biopsy strategy 7	0.2593	0.2424	0.2208	0.1919
Treat all (biopsying any lesion)	0.2088	0.1098	−0.0173	−0.1869
Treat none (biopsying no lesions)	0	0	0	0

*csPCa* clinically significant prostate cancer

Supplementary Table 6 details false negatives and false positives according to different biopsy strategies. Among DLs-based approaches, strategy 7 achieved the smallest number of false negatives (*n* = 8), like strategy 1, and was the only one to miss less aggressive cancers exclusively (ISUP 2 in all cases) at the highest values of specificity and PPV. The second-best DLs-based strategy regarding net benefit values and smaller number of false-negative cases was strategy 6 (8 ISUP 2 cancers and 1 ISUP 4 cancer). PI-RADS 4 was the category that benefited most from using DLs. For example, when using the PI-RADS versus strategy 7, the false positives within PI-RADS 4 category decreased from 89 versus 38 cases (Supplementary Table 6).

## Discussion

In this study, where almost all men underwent multiparametric MRI, we found that using DLs to adjust the PI-RADS version 2.1 categorization of PZ lesions improved the specificity and PPV of the examination. DLs-based biopsy strategies 6 and 7 performed better than strategies 2–4 relying on ADC only. While sensitivity, specificity, PPV, and NPV differences can appear marginal, strategies 6 and 7 showed greater net benefit in shaping biopsy decisions and led to fewer false negatives (mostly ISUP 2 cancers). Strategy 7 was the best overall, achieving increased specificity and PPV compared to PI-RADS categorization (82.9% and 69.2% versus 59.8% and 55.6%) while preserving sensitivity and NPV > 90% and no ISUP ≥ 3 missed cancers. Our results support that DLs help integrate ADCs in image interpretation and reduce the false positives still affecting prostate MRI, especially PI-RADS 4 lesions [19, 22].

To our knowledge, this is the first study investigating DLs in prostate MRI. In line with the well-established relationship between ADC and the Gleason score [9, 23], we observed a significantly negative correlation between DLs and ISUP grading groups (tau −0.614). We also observed that I-DL, L-DL, and VL-DL below 1.03 × 10^−^^3^ mm^2^/s were mostly categorized as PI-RADS 4 or 5. This compares with Gaur et al [24], who found that an ADC cutoff of 1.06 × 10^−^^3^ mm^2^/s highly predicted PI-RADS ≥ 4 categorization. Our results suggest that DLs correlate with imaging and histological findings.

DLs-based strategies 6 and 7 showed greater sensitivity and specificity than pooled values reported in metanalyses for ADC alone (78–80% and 76.9–77%) [25, 26] or the ADC ratio (80% and 80%) [25]. Our results may be explained by DLs being less sensitive than ADC and ADC ratio to significant changes in malignant-to-benign categorization (and vice-versa), which can occur with small value shifts around a single reference threshold. Conversely, DLs compare lesion ADC against various reference ranges linked to specific csPCa risks and ISUP grades, offering a more nuanced approach to capture ADC variability across csPCa aggressiveness than relying on the “Holy Grail” of an absolute ADC or ADC ratio cutoff. Our hypothesis is supported by the fact that in our series, VL-DL, L-DL, I-DL, and H-DL more frequently corresponded to ISUP ≥ 3 cancer, ISUP 2 cancer, benign lesions or ISUP 1 cancer, and benign lesions, respectively. Additionally, DLs simplify ADC measurements by eliminating the need for an additional ROI on a hard-to-standardized reference region.

The most comparable studies are by Moraes et al [27] and Jordan et al [28], who retrospectively adjusted PI-RADS version 2 using ADC (threshold < 0.75 × 10^–3^ mm^2^/s) and ADC ratio (threshold < 0.62) [27], or different ADC thresholds [28]. While Jordan et al proved a significant increase in areas under the curve for 132 PZ lesions and 118 transition zone (TZ) lesions analyzed separately, they did not report sensitivity, specificity, or predictive value variations. Moraes et al showed increased specificity in 91 men (84.9% for ADC and 86.5% for ADC ratio) versus PI-RADS ≥ 3 category as a threshold to auction biopsy (59.9%). In line with our findings, the authors’ results emphasize that the main expected contribution from ADC-related information is to reduce false positives. Differently from us, those Authors found a decrease in sensitivity in detecting Gleason score ≥ 7 (3 + 4 or 4 + 3), which dropped from 97.4% to 70.3% (ADC) and 64.9% (ADC ratio), respectively. A potential explanation relies on using a single ADC threshold for PZ and TZ lesions, while well-known differences in zonal ADC values [27] reasonably require differentiated cutoffs. This indirectly suggests the need for future studies defining TZ-specific DLs and their role within DWI as a non-dominant sequence.

We must acknowledge several study limitations. First, unlike previous breast MRI studies, DLs in our series did not derive from reference values from a metanalysis [16] or a larger multicentric dataset [18]. Source data were heterogeneous in nature, exemplified by the inter-center differences in MRI scanners, sets of b-values to build the ADC map (see Supplementary Tables 1, 2), populations, prostate biopsy route (transrectal versus transperineal), and distribution of PI-RADS v2.1 categories reasonably reflecting the limited inter-agreement of the system [29]. Second, we did not assess the reliability and reproducibility of ADC measurements on an intra- and inter-center basis. Third, the retrospective study design prevented targeting prostate biopsies to PI-RADS 1 and PI-RADS 2 lesions (assuming this was ethical). This made the correlation with the measured ADC only indirect through systemic biopsies taken in adjacent prostate quadrant. While we acknowledge the need for a more direct correlation for these lesions, we believe this had a reasonably limited impact on our results, as the ADC was not intended to trigger prostate biopsies in PI-RADS 1–2 cases, regardless of the biopsy strategy we postulated. Of note, men with PI-RADS 1–2 and without clinical risk factors were not included in the study as they did not undergo systematic biopsy. While this strategy reflects current recommendations and is associated with little risk of missed cancers [3], it could have slightly overestimated the sensitivity and NPV. Finally, we observed a prevalence of csPCa at the lower limit of the expected 30–50% range [22], particularly within the PI-RADS 5 category (67.5%). While comparable to that previously reported using the PI-RADS version 1 (69%) [30], our finding is lower than the 74% prevalence found on a per-lesion basis in a PI-RADS version 2.1-centered metanalysis [31]. We cannot exclude overcalls of PI-RADS 5 in our series and, in turn, overinflation of strategy 7 performance in avoiding prostate biopsy in PI-RADS 5 lesions associated with H-DL.

Taken together, these limitations emphasize the risk of limited reproducibility and overfitting of our results and, consequently, the impossibility of claiming that the DLs we found can be “the definitive” in terms of ADC range values, inherent risk of csPCa, and impact on PI-RADS-related biopsy decisions. However, at this stage, our aim was not to validate a system for large-scale use but to demonstrate that DLs are feasible in prostate MRI and can improve biopsy decisions by enhancing specificity and PPV. Despite heterogeneity, we matched those proof-of-concept goals and reasonable proof of robustness of the ADC values, given their stand-alone sensitivity and specificity of 93.4% and 75.5%. Further studies should refine the ADC ranges of DLs, assess the tolerance to unavoidable inter-center differences, evaluate whether DLs can apply to the transition zone, directly compare them to strategies such as the ADC ratio, and assess whether DLs can comparably improve the diagnostic performance when performing unenhanced prostate MRI.

In conclusion, DLs can be derived from prostate MRI. Combining them with the PI-RADS version 2.1 translated into increased per-lesion specificity and PPV for csPCa of the PZ, unaltered sensitivity and NPV, and greater net benefit in shaping biopsy decisions than ADC-based strategies or the PI-RADS alone. DLs are promising as a tool to incorporate the ADC value into prostate MRI interpretation.

## Supplementary information

ELECTRONIC SUPPLEMENTARY MATERIAL

## Abbreviations

ADC: Apparent diffusion coefficient
csPCa: Clinically significant prostate cancer
DLs: Diffusion levels
DWI: Diffusion-weighted imaging
DRE: Digital rectal examination
EUSOBI: European Society of Breast Imaging
H-DL: High DL
I-DL: Intermediate DL
ISUP: International Society of Urological Pathology
L-DL: Low DL
NPV: Negative predictive value
PZ: Peripheral zone
PPV: Positive predictive value
PI-RADS: Prostate imaging reporting and data system
PSA: Prostatic specific antigen
ROC: Receiving operating characteristics
ROI: Region of interest
VL-DL: Very low DL
