# Supplementary material for: Diffusion levels for quantitative assessment of the apparent diffusion coefficient value in prostate MRI: a proof-of-concept bicentric study
Source: Eur Radiol. 2025 Apr 7;35(10):6171–82. doi: 10.1007/s00330-025-11547-8 (PMC12417251; doi:10.1007/s00330-025-11547-8)
Supplement: Supplementary file 1 — ELECTRONIC SUPPLEMENTARY MATERIAL [file 330_2025_11547_MOESM1_ESM.pdf]

Diffusion levels for quantitative assessment of the apparent diffusion coefficient value in prostate MRI: a proof-of-concept bicentric study

ELECTRONIC SUPPLEMENTARY MATERIAL

Supplementary Fig. 1 - Box-and-whisker plot showing the distribution of the apparent diffusion coefficient (ADC) values of benign lesions, International Society of Urogenital Pathology (ISUP) 1 cancers and ISUP ≥2 cancers. The reported p values refer to pairwise comparisons

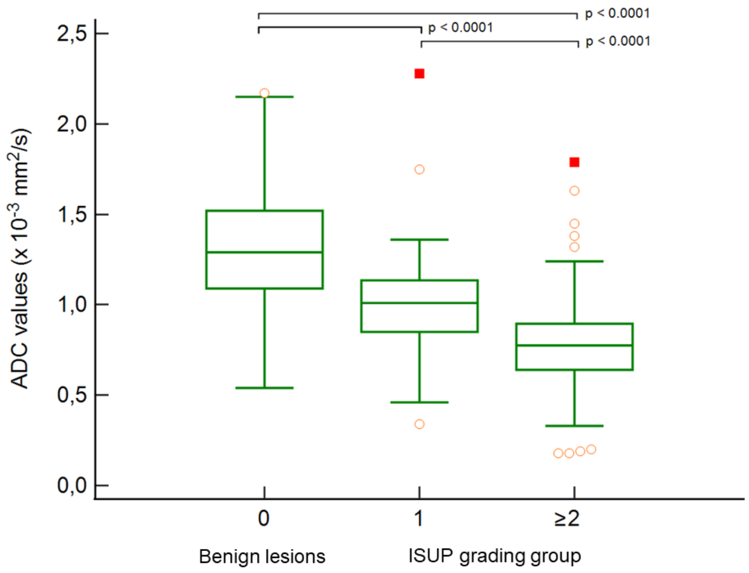

Supplementary Fig. 2 – Receiver operating characteristic (ROC) curve of the ADC in assessing ISUP ≥2 cancers in the study cohort, with related area under the curve (AUC).

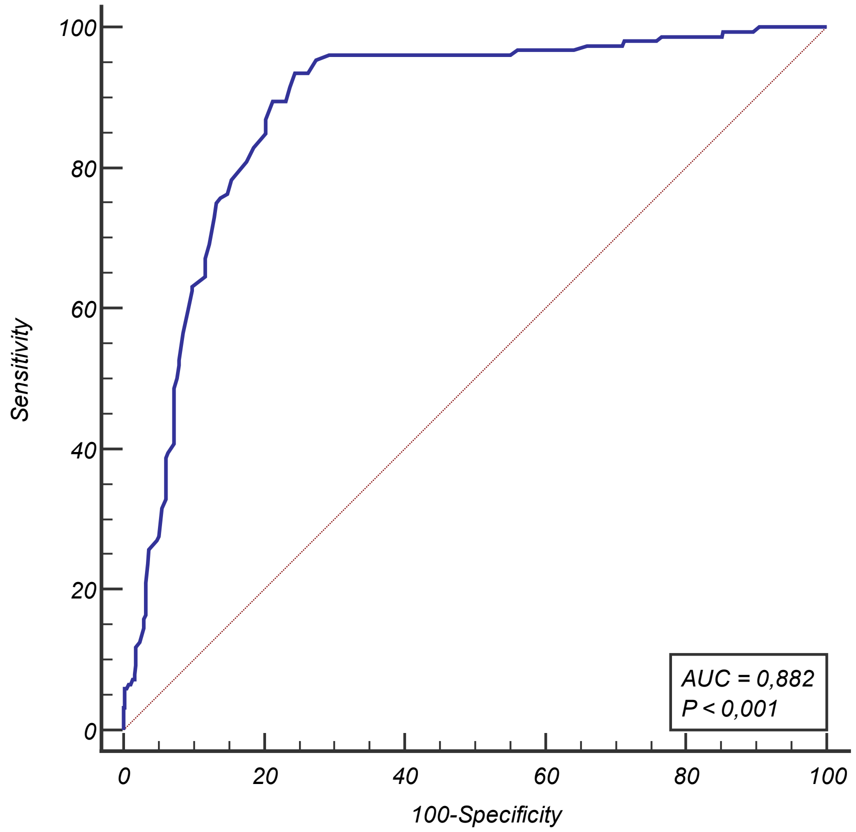

	DWI sequences		T2-weighted imaging	DCE
	First sequence*	Second sequence**		
**Sequence design**	SS-EPI	SS-EPI	TSE	THRIVE
**Weighting**	DWI	DWI	T2	T1
**Acquisition plane**	Transverse	Transverse	Transverse/coronal/sagittal (at least two planes)	Transverse
**TR (ms)**	5350	5424	4727/3076/3714	3.5
**TE (ms)**	68	78	80/80/80	1.77
**Echo train length**	-	-	8/16/16	-
**EPI factor**	109	109	-	-
**Half scan factor**	0.62	0.62	no	no
**FOV (mm x mm)**	200 x 200	200 x 200	180x180/180x180/180x180	200x200
**Acquisition voxel size**	2 x 2 x 3	2 x 2 x 3	0.6 x 0.6 x 3	1.2 x 1.2 x 8
**Reconstruction pixel size (mm x mm x mm)**	1.4 x 1.4 x 3	1.4 x 1.4 x 3	0.45 x 0.45 x 3	0.63 x 0.63 x 4
**Number of slices**	24	24	24/20/20	20
**Interslice gap (mm)**	0	0	0/0/0	0
**b-values (s/mm^2)/number of excitations**	100/1, 500/1, 1000/2	100/1, 2000/3	-	-
**Number of excitations**	-	-	2/1/1	1
**Fat saturation**	SPAIR	SPAIR	-	Spectral fat saturation
**Parallel imaging (x acceleration factor)**	SENSE x 2	SENSE x 2	SENSE x 2.5/1/1	SENSE x 2
**Acquisition time (min)**	3.2	3.2	5.3/4.2/5.1	Total acquisition time 6 (0.14 x 44 acquisitions)

* Used to build the apparent diffusion coefficient map by fitting signal intensity versus the b-values up to 1000 s/mm^2.

** Used to provide the maximum b-value images (b = 2000 s/mm^2).

Supplementary Tab. 1 - Acquisition parameters of multiparametric prostate magnetic resonance imaging acquired in Center 1. Men were asked to self-administer a rectal enema 1 hour to 30 minutes before the examination. Preparation also included intramuscular or intravenous administration of 20 mg of hyoscine-butyl-bromide as an antiperistaltic agent before entering the magnet room or directly on the MRI table. In the diffusion-weighted sequence, the time of echo was set as the minimum possible, while diffusion gradients were oriented along the three main space directions. DWI = diffusion-weighted imaging; TSE-T2WI = turbo spin echo T2-weighted imaging; DCE = dynamic contrast-enhanced; THRIVE = T1-weighted high-resolution isotropic volume examination; SS-EPI = single-shot echo-planar imaging; TR = time of repetition; EPI = echo-planar imaging; TE = time of echo; FOV = field of view; MRI = magnetic resonance imaging; SENSE = sensitivity encoding; SPAIR = spectral adiabatic inversion recovery

	DWI sequences		T2-weighted imaging	DCE
	First sequence*	Second sequence**		
**Sequence design**	SS-EPI	RS-EPI	TSE	Flash 3D/VIBE
**Weighting**	DWI	DWI	T2	T1
**Acquisition plane**	Transverse	Transverse	Transverse/coronal/sagittal (at least two planes)	Transverse
**TR (ms)**	>5000	>5000	>3000 each	<9
**TE (ms)**	<90	<90	78-101 each	Lowest possible
**Echo train length**	-	-	8-16	-
**EPI factor**	<100	110-159	-	-
**Half scan factor**			No	No
**FOV (mm x mm)**	200x200/210-210	200x200	180-200 each	200-260x200-260
**Acquisition voxel size**	1.2-1.6x1.2-1.6x3	1.3-2x1.3-2x3-4	0.6 x 0.6 x 3	1.2-1.4 x 1.2-1.4 x 3.5
**Reconstruction pixel size (mm x mm x mm)**	Same as above	Same as above	Same as above	Same as above
**Number of slices**	20-24	20-24	20-24	25-34
**Interslice gap (mm)**	0	0	0	0
**b-values (s/mm^2)/number of excitations**	0-50, 1000	0-50, 1000-1600	-	-
**Number of excitations**	-	-	1-2	1
**Fat saturation**	SPAIR	SPAIR	-	No fs, SPAIR, Dixon
**Parallel imaging (x acceleration factor)**	GRAPPA x 2, SMS 2	GRAPPA x 2, SMS 2	GRAPPA 2-3	GRAPPA x 2-3
**Acquisition time (min)**	2:56-4:14	3:30-5:48	2:08-3:20	Total acquisition time 2-4 min (0.11-0.14 temporal resolution)

* Used to build the apparent diffusion coefficient map by fitting signal intensity versus the b-values up to 1000 s/mm^2.

** Used to provide the maximum b-value images (b up to 1600 s/mm^2).

Supplementary Tab. 2 - Acquisition parameters of multiparametric prostate magnetic resonance imaging acquired in Center 2. Patient preparation was the same as in Center 1 (see Supplementary Tab. 1). In the diffusion-weighted sequence, the time of echo was set as the minimum possible, while diffusion gradients were oriented along the three main space directions. GRAPPA = GeneRalized Autocalibrating Partially Parallel Acquisitions; RS-EPI = readout segmented echoplanar imaging. The abbreviations are similar to those in Supplementary Tab. 1.

	Center 1	Center 2
**Men**	189	72
**Men with prior negative biopsy***	168	62
**Men with family history of prostate cancer**	21	10
**Lesions**	255	273
**Age<br>Median<br>IQR**	65<br>(59-70)	68<br>(61-73)
**PSA<br>Median<br>IQR**	6.05<br>(4.63-8.38)	7.12<br>(5.17-10.88)
**PSAD<br>Median<br>IQR**	0.12<br>(0.08-0.16)	0.18<br>(0.10-0.31)
**PI-RADS (%; IC 95%)**		
**1**	35/255<br>(13.7%; 9.7-18.3)	13/273<br>(4.8%; 2.3-7.3)
**2**	59/255<br>(23.2%; 17.8-28.2)	126/273<br>(46.2%; 40.1-51.9)
**3**	20/255<br>(7.8%; 72.9-83.1)	20/273<br>(7.3%; 4.2-10.4)
**4**	109/255<br>(42.7%; 36.9-46.1)	66/273<br>(24.1%; 18.9-29.1)
**5**	32/255<br>(12.6%; 8.9-17.1)	48/273<br>(17.6%; 13.1-22.1)
**ISUP grading group (%; IC 95%)**		
**No cancer**	127/255<br>(49.8%; 43.7-55.9)	158/273<br>(57.9%; 52.0-63.8)
**1**	53/255<br>(20.8%; 15.8-25.8)	38/273<br>(13.9%; 9.8-18.0)
**2**	38/255<br>(14.9%; 10.5-19.3)	34/273<br>(12.5%; 8.6-16.4)
**3**	26/255<br>(10.2%; 6.5-13.9)	18/273<br>(6.6%; 3.7-9.5)
**4**	6/255<br>(2.3%; 0.5-4.3)	11/273<br>(4.0%; 1.7-6.4)
**5**	5/255<br>(2%; 0.3-3.7)	14/273<br>(5.1%; 2.5-7.7)

*Performed at least 12 months before MRI.

Supplementary Tab. 3 – Clinical and imaging features of the study patients stratified on a per-center basis. IQR = interquartile range; ISUP = International Society of Urological Pathology; PI-RADS = Prostate Imaging Reporting and Data System version 2.1.

	ISUP						
**DLs**	**0**	**1**	**2**	**3**	**4**	**5**	
**Very low (VL-DL)**	15	20	27	35	15	14	126 (23.9%)
**Low (L-DL)**	11	10	16	6	1	2	46 (8.7%)
**Intermediate (I-DL)**	30	17	22	3	1	3	76 (14.4%)
**High (H-DL)**	229	44	7	0	0	0	280 (53.0%)
	285 (54.0%)	91 (17.2%)	72 (13.6%)	44 (8.3%)	17 (3.2%)	19 (3.6%)	528

Supplementary Tab. 4 – Prevalence of different ISUP grading groups on a per-DL basis. ISUP “0” is meant as “no cancer present”.

	PI-RADS					
**DLs**	**1**	**2**	**3**	**4**	**5**	
**Very low (VL-DL)**	1	6	5	65	49	126 (23.9%)
**Low (L-DL)**	1	5	4	23	13	46 (8.7%)
**Intermediate (I-DL)**	4	18	5	36	13	76 (14.4%)
**High (H-DL)**	42	156	26	51	5	280 (53.0%)
	48 (9.1%)	185 (35.0%)	40 (7.6%)	175 (33.1%)	80 (15.2%)	528

Supplementary Tab. 5 – Prevalence of different PI-RADS categories on a per-DL basis

Biopsy strategy	False-negative findings			False-positive findings		
	Number	ISUP grading group	PI-RADS categorization	Number	ISUP grading group	PI-RADS categorization
**1**	8	ISUP 2 in all cases	PI-RADS 2 in all cases	151	ISUP 1 in 76 cases + Benign lesions in 75 cases	- PI-RADS 3 in 36 cases (9 ISUP1; 27 benign lesions)<br>- PI-RADS 4 in 89 cases (53 ISUP 1; 36 benign lesions)<br>- PI-RADS 5 in 26 cases (14 ISUP 1; 12 benign lesions)
**2**	8	ISUP 2 in all cases	PI-RADS 2 in all cases	122	ISUP 1 in 69 cases + 53 benign lesions	- PI-RADS 3 in 7 cases (2 ISUP 1; 5 benign lesions)<br>- PI-RADS 4 in 89 cases (53 ISUP 1; 36 neg)<br>- PI-RADS 5 in 26 cases (14 ISUP 1; 12 benign lesions)
**3**	13	ISUP 2 in 12 cases + ISUP 3 in 1 case	PI-RADS 2 in 8 cases + PI-RADS 4 in 5 cases	70	ISUP 1 in 42 cases + 28 benign lesions	- PI-RADS 3 in 7 cases (2 IUSP 1; 5 benign lesions)<br>- PI-RADS 4 in 37 cases (26 ISUP 1; 11 benign lesions)<br>- PI-RADS 5 in 26 cases (14 ISUP 1; 12 benign lesions)
**4**	14	ISUP 2 in 13 cases + ISUP 3 in 1 case	PI-RADS 2 in 8 cases + PI-RADS 4 in 5 cases + PI-RADS 5 in 1 case	64	ISUP 1 in 42 cases + 23 benign lesions	- PI-RADS 3 in 7 cases (2 ISUP 1; 5 benign lesions)<br>- PI-RADS 4 in 37 cases (26 ISUP 1; 11 benign lesions)<br>- PI-RADS 5 in 20 cases (13 ISUP 1; 7 benign lesions)
**5**	28	ISUP 2 in 22 cases + ISUP 3 in 2 cases + ISUP 4 in 2 cases + ISUP 5 in 2 cases	PI-RADS 2 in 8 cases + PI-RADS 3 in 1 case + PI-RADS 4 in 19 cases	49	ISUP 1 in 28 cases + 21 benign lesions	- PI-RADS 3 in 2 cases (benign lesions)<br>- PI-RADS 4 in 21 cases (14 ISUP 1; 7 benign lesions)<br>- PI-RADS 5 in 26 cases (14 ISUP 1; 12 benign lesions)
**6**	9	ISUP 2 in 8 cases + ISUP 4 in 1 case	PI-RADS 2 in 8 cases + PI-RADS 3 in 1 case	66	ISUP 1 in 40 cases + 26 benign lesions	- PI-RADS 3 in 2 cases (benign lesions)<br>- PI-RADS 4 in 38 cases (26 ISUP 1; 12 benign lesions)<br>- PI-RADS 5 in 26 cases (14 ISUP 1; 12 benign lesions)
**7**	8	ISUP 2 in all cases	PI-RADS 2 in all cases	64	ISUP 1 in 41 cases + 23 benign lesions	- PI-RADS 3 in 5 cases (2 ISUP 1; 3 benign lesions)<br>- PI-RADS 4 in 38 cases (26 ISUP 1; 12 benign lesions)<br>- PI-RADS 5 in 21 cases (13 ISUP 1; 8 benign lesions)

Supplementary Tab. 6 – Number, ISUP grading group and PI-RADS version 2.1 categorization of the false-negative and false-positive categorizations made with different biopsy strategies (see the main text for definition)
